# Biomechanical symmetry in elite rugby union players during dynamic tasks: an investigation using discrete and continuous data analysis techniques

**DOI:** 10.1186/s13102-015-0006-9

**Published:** 2015-06-19

**Authors:** Brendan Marshall, Andrew Franklyn-Miller, Kieran Moran, Enda King, Chris Richter, Shane Gore, Siobhán Strike, Éanna Falvey

**Affiliations:** 1 Sports Medicine Department, Sports Surgery Clinic, Santry Demesne, Dublin, Ireland; 2 School of Health and Human Performance, Dublin City University, Dublin, Ireland; 3 Department of Life Sciences, Roehampton University, London, UK; 4 Centre for Health, Exercise and Sports Medicine, University of Melbourne, Melbourne, Australia; 5 Insight Centre for Data Analytics, Dublin City University, Dublin, Ireland; 6 Department of Medicine, University College Cork, Cork, Ireland

**Keywords:** Landing, Cutting, Dominant versus non-dominant, Kinetics, Kinematics

## Abstract

**Background:**

While measures of asymmetry may provide a means of identifying individuals predisposed to injury, normative asymmetry values for challenging sport specific movements in elite athletes are currently lacking in the literature. In addition, previous studies have typically investigated symmetry using discrete point analyses alone. This study examined biomechanical symmetry in elite rugby union players using both discrete point and continuous data analysis techniques.

**Methods:**

Twenty elite injury free international rugby union players (mean ± SD: age 20.4 ± 1.0 years; height 1.86 ± 0.08 m; mass 98.4 ± 9.9 kg) underwent biomechanical assessment. A single leg drop landing, a single leg hurdle hop, and a running cut were analysed. Peak joint angles and moments were examined in the discrete point analysis while analysis of characterising phases (ACP) techniques were used to examine the continuous data. Dominant side was compared to non-dominant side using dependent t-tests for normally distributed data or Wilcoxon signed-rank test for non-normally distributed data. The significance level was set at α = 0.05.

**Results:**

The majority of variables were found to be symmetrical with a total of 57/60 variables displaying symmetry in the discrete point analysis and 55/60 in the ACP. The five variables that were found to be asymmetrical were hip abductor moment in the drop landing (*p* = 0.02), pelvis lift/drop in the drop landing (*p* = 0.04) and hurdle hop (*p* = 0.02), ankle internal rotation moment in the cut (*p* = 0.04) and ankle dorsiflexion angle also in the cut (*p* = 0.01). The ACP identified two additional asymmetries not identified in the discrete point analysis.

**Conclusions:**

Elite injury free rugby union players tended to exhibit bi-lateral symmetry across a range of biomechanical variables in a drop landing, hurdle hop and cut. This study provides useful normative values for inter-limb symmetry in these movement tests. When examining symmetry it is recommended to incorporate continuous data analysis techniques rather than a discrete point analysis alone; a discrete point analysis was unable to detect two of the five asymmetries identified.

**Electronic supplementary material:**

The online version of this article (doi:10.1186/s13102-015-0006-9) contains supplementary material, which is available to authorized users.

## Background

The assessment of movement control and inter-limb symmetry during functional tasks is increasingly popular as a means of screening for predisposition to injury, in the evaluation of athletic performance and in the assessment of rehabilitation following injury [1–3]. A number of research studies provide support for these practises, and in turn, the premise that functional asymmetry (side to side differences in kinetics or kinematics) [4] may provide an insight into future injury risk [5–7].

Various studies have identified kinetic and kinematic asymmetry as an underlying risk factor for injury. Hewett and colleagues [7] found significantly greater asymmetries in landing knee abduction moments (6.4 times greater) in individuals who went on to injure their anterior cruciate ligament. In another prospective study, Paterno and colleagues [8] found that individuals who suffered a second anterior cruciate ligament injury had 4.1 times greater asymmetry in knee extensor moments on landing.

Asymmetry as an injury risk factor is not confined to a single joint, variable or injury type. Angle and moment variables at the ankle [9, 10], knee [7, 11], hip [8, 12], pelvis [13] and torso [14], as well as ground reaction forces [15] and ground contact times [16] have all been implicated in the development of lower extremity injury. Such injuries include ankle ligament injury [10], tibial stress fracture [11], knee ligament injury [8] and patellofemoral pain syndrome [17]. It is suggested that a notable asymmetry in these biomechanical factors may increase the risk of lower extremity injury in one limb over the other [7, 6].

In order to use measures of asymmetry as a means of identifying individuals predisposed to injury it is extremely important to establish normative values for uninjured individuals on a number of biomechanical measures. Normative values across multiple joints are not only required due to the numerous factors associated with injury, but also because poor movement control and excessive force at a proximal/distal joint can influence moments and forces at another joint [13, 18]. Zazulak and colleagues [14], for example, found that deficits in neuromuscular control at the trunk could prospectively predict knee injury risk. This phenomenon arises due to the inter-linked nature of the body’s segments and the presence of bi-articular muscles (intersegmental movement constraint).

While some normative values of asymmetry exist for straight line running [6, 19], and bilateral landing [20], a full range of three dimensional measures on more specific multi-directional tasks, such as uni-lateral landing, hopping and cutting, are lacking in the literature. These more dynamic tasks are commonly associated with injury [5, 21–23]. In addition, there is a need for normative symmetry values for elite athletic populations as the majority of previous work in this area has been carried out with sub-elite athletes [6, 24, 25]. Elite athletes may develop asymmetries due to the preferential use of a dominant limb in training. Vittasalo and colleagues [26], for example, highlighted that training history influences the timing and magnitude of lower extremity muscle activation on landing in a jump.

Previous studies investigating biomechanical symmetry in dynamic movements have typically done so using discrete points (e.g. peak values) [20, 24, 25]. There are a number of limitations with this type of analysis however: (a) asymmetry may occur over phases that are not captured in a single data point, (b) the timing of discrete points can differ between limbs, and (c) the discrete points utilised typically vary between studies [27]. Continuous data analysis techniques [28], such as Analysis of Characterising Phases (ACP) [27], have been developed to overcome these issues but it appears that a comparison of symmetry findings from both continuous and discrete analyses has yet to be undertaken for dynamic sporting movements. Such an examination is warranted as the use of a discrete point analysis alone may not detect all significant asymmetries.

The primary aim of this study was to examine biomechanical symmetry during multi directional neuromuscular challenge tests in a cohort of elite injury free rugby union players. It was hypothesised that there would be a general trend toward inter-limb symmetry but that some biomechanical variables would display asymmetry due to the preferential use of a dominant limb in training. A secondary aim was to compare the findings of both discrete point and ACP analyses techniques. It was hypothesised that the results of these distinct analyses would differ due to the utilisation of discrete point and continuous data, respectively. In an attempt to adequately simulate movements that are associated with injury in field sport play [5], a single-leg landing [29], a single-leg lateral hop [5], and a change-of-direction cut [21] were examined.

## Methods

### Participants

Prior to the commencement of the rugby season, twenty elite rugby union players (mean ± SD: age 20.4 ± 1.0 years; height 1.86 ± 0.08 m; mass 98.4 ± 9.9 kg) were recruited to undergo three dimensional (3D) biomechanical assessment. All participants were professional academy players (*n* = 11 had made senior club appearances), and all had international caps at an age-group level. Both forward (*n* = 11) and back (*n* = 9) players were selected and all were injury free for three months at the time of testing and had no history of chronic lower extremity injury or surgery in the previous two years (self-report). The study was approved by the Sport Surgery Clinic Hospital Ethics Committee and all subjects signed informed consent.

### Experimental protocol

Prior to testing, participants’ mass and height was recorded using an electronic scale (Seca 876) and stadiometer (Seca 213) and their dominant leg was identified (the leg one would use to kick a ball for distance). A warm-up consisting of a three minute treadmill jog at 8 km/h followed by five body weight bilateral squats was then undertaken. Testing involved three trials of: (1) a single leg drop landing, (2) a single leg hurdle hop, and (3) a running cut. The 3D Biomechanics Laboratory is equipped with an artificial grass surface (polyethylene mono filament, Condor Grass, Holland) which is permanently and firmly fixed to the force plates (Sanctuary Synthetic Adhesive, Ireland). Participants wore their own molded football boots.

**Fig. 1:**
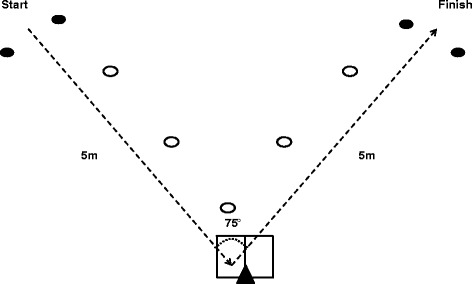
Layout for a right footed plant and cut left. From a standing start participants sprinted maximally toward a marker placed on the floor, made a single complete foot contact on the force plate, and performed a 75° cut before sprinting maximally to the finish

The drop landing was initiated from a 30 cm step where participants stood upright with their hands across their chest and their non-weight bearing foot behind with an approximate 90° knee bend. They then dropped off the step, made a uni-lateral landing on the force platform and held the landing position for 2 s [30]. An additional movie file shows this in more detail [see Additional file 1]. Participants were instructed to drop directly from the 30 cm height rather than jump vertically. The hurdle hop consisted of a lateral hop over a 15 cm hurdle and an immediate hop back to the initial starting position. The distance between foot contacts was approximately 40 cm; the distance between force plate centres. Participants undertook the hop as quickly as possible, and while the free leg was in the same orientation as described for the drop landing, the arms were free to move [see Additional file 2]. The landing from the first hop over the hurdle was analysed. For the cut, participants ran as fast as possible toward a marker placed on the floor, made a single complete foot contact on the force plate, and performed a 75° cut before running maximally to the finish (Fig. 1). An additional movie file shows the cut in greater detail [see Additional file 3]. Time to complete the cut was recorded using the Hotspot timing system (Games Education - Hotspot, UK).

Additional file 1:**Drop landing clip.** Video clip of the drop landing movement test.Additional file 2:**Hurdle hop clip.** Video clip of the hurdle hop movement test.Additional file 3:**Running cut clip.** Video clip of the running cut movement test.

Testing was carried out in the order of drop landing, hurdle hop and cut and all trials of one movement were completed on one leg (the choice of leg was randomised) before moving to the other leg. Participants undertook two practice trials of each movement (submaximal practice trials for the cut) before capture. Recovery of 30s was allocated between repetitions of the drop landing and hop with 1 min allocated between trials of the cut. To facilitate an assessment of the test-retest reliability of measures, fifteen players were re-tested one week after their initial testing session.

### Data acquisition and analysis

An eight camera 3D motion analysis system (Vicon - Bonita B10, UK), synchronized with two 40x60cm force platforms (AMTI – BP400600, USA), was used to collect movement data. The force platforms had force ranges in the Fx, Fy and Fz directions of 2224 N, 2224 N and 4448 N, respectively and were zeroed at the start of every new data capture session. Force plate calibration was checked by placing a known weight on the plates and examining the subsequent data. Reflective markers (1.4 cm diameter) were placed at bony landmarks on the lower limbs, pelvis and trunk according to Plug in Gait marker locations [31]. Vicon Nexus software controlled simultaneous collection of motion and force data at 200Hz and 1,000Hz, respectively and both were filtered using a fourth order Butterworth filter with a cut-off frequency of 15Hz to avoid impact artefacts [32, 33]. The Vicon Plug in Gait modelling routine defined rigid body segments (foot, shank, thigh, pelvis and torso) and used standard inverse dynamics techniques [34] to calculate segmental and joint kinematics and kinetics.

Ankle, knee, hip, pelvis and thorax angles were calculated as well as internal joint moments at the hip, knee and ankle during foot contact with the force plate. Peak ground reaction forces and ground contact time in the cut were also examined. These variables were chosen as they have previously been associated with the development of numerous lower extremity injuries [7–16].

Angles were normalised to a standing static trial [35] and thorax angles were calculated relative to the pelvis as opposed to the global axis. It was not possible to measure thorax angles in the drop landing due to upper body marker occlusion. Transverse plane angles and moments for the single leg drop landing and hurdle hop were calculated but for brevity are not reported. The drop landing and hurdle hop involve movement primarily in the sagital and frontal plane, and no significant inter-limb differences in transverse plane variables were observed in these tasks. Similarly, medial/lateral and longitudinal ground reaction forces in the hurdle hop and drop landing were captured but are not reported; these measures displayed no inter-limb asymmetries.

For the discrete point analysis, peak variable values were calculated during nominal eccentric and concentric phases (eccentric phase only in the drop landing). Initial contact with the force platform marked the start of the eccentric phase in all movements. The minimum vertical height of the centre-of-mass marked the end of the eccentric phase in the drop landing while the maximal lateral/anterior position of the centre-of-mass was used to identify the end of the eccentric/start of the concentric phase in the hop and cut, respectively. The end of the concentric phase in the hop and cut occurred at toe-off from the force platform. Discrete-point data from the eccentric phase, which is more typically associated with injury development [6, 36], is presented herein while data for the concentric phase of the hurdle hop and drop landing is presented as additional data [see Additional file 4: Table S1 and Additional file 5: Table S2, respectively]. The mean of each participant’s three trials for each limb was utilised in further analysis.

For the continuous waveform analysis, Analysis of Characterising Phases (ACP) was utilised; ACP has previously been shown to be effective at identifying additional features in biomechanical data to those identified in a discrete point analysis [27]. ACP was performed as described in Richter and colleagues [37] and landmark registration was applied to reduce phase shift intra subject variability [37]. As with the discrete point analysis, the mean of each participant’s three trials was utilised for further analysis.

### Statistical analysis

For both the discrete point analysis and ACP a Levene's test and a Kolmogorov-Smirnov test was used to examine equality of variance and normality of distribution, respectively. If data were parametric a paired Student's *t*-test was used to examine differences between the dominant and non-dominant sides [20], while a Wilcoxon signed-rank test was otherwise performed. It was assumed that an asymmetry existed when a significant between limb difference was found [20].

As a further measure of asymmetry an absolute asymmetry index was also calculated as per Karaminidis and colleagues [19] [Eq. 1] for the discrete point data. The asymmetry index is a popular measure that is often cited in the literature [38] but its ability to provide a standardised score across variables of different magnitudes has been questioned [24].

1 $$ \mathrm{Asymmetry}\ \mathrm{Index}\ \% = \frac{\left|{\mathrm{X}}_{\mathrm{D}}\hbox{-} {\mathrm{X}}_{\mathrm{ND}}\right|}{0.5\left(\ {\mathrm{X}}_{\mathrm{D}} + {\mathrm{X}}_{\mathrm{ND}}\right)}\kern1em *\;100 $$

where X_D_ is the measure of the dominant side; X_ND_ is the measure of the non-dominant side.

The authors deemed it inappropriate to calculate an asymmetry index for the continuous data; the use of a single value to represent differences between two continuous data sets would be subject to the limitations of a discrete analysis that we were attempting to avoid.

An intraclass correlation coefficient (ICC (3,k)) was used to examine the test-retest reliability of peak values for each variable. The ICC classifications of Ford and colleagues [39] (<0.4 poor, 0.4–0.75 fair to good, >0.75 excellent) were employed to describe the range of values obtained.

The significance level was set at α = 0.05. Data processing and statistical analyses were performed using MATLAB (R2012a, MathWorks Inc., USA).

## Results

Discrete point findings for the drop landing, hurdle hop and cut are displayed in Tables 1, 2 and 3, respectively. Peak variable magnitudes, asymmetry index and the findings of tests of significant difference between dominant and non-dominant sides (with effect sizes) are presented. The vast majority of variables displayed no statistically significant asymmetries (p > 0.05) in the drop landing (14/15), hurdle hop (16/17) and cut (27/28). Asymmetry indexes for these variables however ranged from 0 to 143 % in the drop landing, 1–264 % in the hurdle hop and 1–49 % in the cut.

**Table 1:** Drop landing discrete point findings – inter-limb differences in peak variable magnitudes during the eccentric phase

Variable	Dominant	Non-dominant	Diff	AI%	*p* value	Effect size
Ankle angles (deg)						
DorsiF (+)/PlantF(−)	18.4 ± 2.8	19.4 ± 3.8	1.0	5	0.46	0.28
Ever(+)/ Inv(−)	5.7 ± 2.4	5.0 ± 2.2	0.7	17	0.39	−0.32
Ankle moments (Nm/kg)						
PlantF(+)/DorsiF(−)	2.7 ± 0.4	2.8 ± 0.6	0.1	4	0.39	0.32
Ever(+)/ Inv(−)	−0.1 ± 0.2	−0.2 ± 0.2	0.1	67	0.52	−0.24
Knee angles (deg)						
Flex(+)/Ext(−)	66.6 ± 8.8	66.3 ± 8.0	0.3	1	0.93	−0.03
Var(+)/Valg(−)	4.3 ± 5.6	7.6 ± 8.5	3.3	143	0.22	0.46
Knee moments (Nm/kg)						
Ext (+)/Flex(−)	3.1 ± 0.4	3.1 ± 0.3	0.0	0	0.95	0.02
Valg(+)/Var(−)	1.9 ± 0.4	2.0 ± 0.5	0.1	5	0.56	0.22
Hip angles (deg)						
Flex(+)/Ext(−)	59.3 ± 10.9	59.4 ± 9.1	0.1	0	0.98	0.01
Add(+)/ Ab(−)	9.3 ± 5.6	10.0 ± 3.0	0.7	19	0.70	0.15
Hip moments (Nm/kg)						
Ext (+)/Flex(−)	5.4 ± 2.0	5.0 ± 1.3	0.4	8	0.47	−0.27
Ab(+)/Add(−)	2.7 ± 0.7	2.2 ± 0.8	0.5	20	0.09	−0.63
Pelvis angles (deg)						
AntT(+)/PostT(−)	13.8 ± 8.0	14.5 ± 7.5	0.7	8	0.79	0.10
Contra Drop(+)/Contra Lift(−)	−12.1 ± 4.0	−8.9 ± 3.4*	3.2	31	0.02	0.80
Ground reaction force (N/kg)						
Vertical	43.7 ± 5.1	44.8 ± 6.6	1.1	3	0.61	0.19

*Significant inter-limb difference (*p* < 0.05)

Diff: difference; AI: asymmetry index; Sig: significance

DorsiF: dorsiflexion; PlantF: plantarflexion; Ever: eversion; Inv: inversion; Flex: flexion; Ext: extension; Var: varus; Val: valgus; Add: adduction; Ab: abduction; AntT: anterior tilt; PostT: posterior tilt; Contra: contralateral

**Table 2:** Hurdle hop discrete point findings – inter-limb differences in peak variable magnitudes during the eccentric phase

Variable	Dominant	Non-dominant	Diff	AI%	*p* value	Effect size
Ankle angles (deg)						
DorsiF (+)/PlantF(−)	16.8 ± 4.2	17.8 ± 4.4	1.0	5	0.58	0.21
Ever(+)/ Inv(−)	4.5 ± 2.4	4.2 ± 2.6	0.3	8	0.73	−0.13
Ankle moments (Nm/kg)						
PlantF(+)/DorsiF(−)	3.4 ± 0.5	3.4 ± 0.5	0.0	0	0.86	0.07
Ever(+)/ Inv(−)	0.4 ± 0.2	0.4 ± 0.2	0.0	0	0.93	0.04
Knee angles (deg)						
Flex(+)/Ext(−)	42.3 ± 10.3	43.3 ± 8.8	1.0	2	0.79	0.10
Var(+)/Valg(−)	−3.1 ± 5.6	−0.6 ± 5.7	2.5	132	0.25	0.44
Knee moments (Nm/kg)						
Ext (+)/Flex(−)	2.6 ± 0.7	2.8 ± 0.5	0.2	7	0.50	0.26
Valg(+)/Var(−)	1.9 ± 0.6	2.1 ± 0.6	0.2	10	0.23	0.46
Hip angles (deg)						
Flex(+)/Ext(−)	34.0 ± 6.5	33.3 ± 7.2	0.7	2	0.79	−0.10
Add(+)/ Ab(−)	−8.1 ± 5.3	−5.9 ± 4.0	2.2	31	0.24	0.45
Hip moments (Nm/kg)						
Ext (+)/Flex(−)	2.9 ± 1.0	2.9 ± 0.9	0.0	0	1.00	0.00
Ab(+)/Add(−)	1.5 ± 0.3	1.5 ± 0.4	0.0	0	0.55	0.23
Pelvis angles (deg)						
AntT(+)/PostT(−)	11.9 ± 4.4	11.7 ± 4.3	0.2	2	0.91	−0.05
Contra Drop(+)/Contra Lift(−)	−1.4 ± 4.7	3.1 ± 4.1*	4.5	264	0.01	0.92
Thorax angles (deg)						
Flex(+)/Ext(−)	6.8 ± 7.9	4.7 ± 7.4	2.1	38	0.46	0.29
LatFlex(+)/MedFlex(−)	7.9 ± 5.9	8.7 ± 4.0	0.8	10	0.68	0.16
Ground reaction force (N/kg)						
Vertical	29.2 ± 4.0	28.6 ± 2.6	0.6	2	0.67	0.16

*Significant inter-limb difference (*p* < 0.05)

Diff: difference; AI: asymmetry index; Sig: significance

DorsiF: dorsiflexion; PlantF: plantarflexion; Ever: eversion; Inv: inversion; Flex: flexion; Ext: extension; Var: varus; Val: valgus; Add: adduction; Ab: abduction; AntT: anterior tilt; PostT: posterior tilt; Contra: contralateral; LatFlex: lateral flexion; MedFlex: medial flexion

**Table 3:** Running cut discrete point findings – inter-limb differences in peak variable magnitudes during the eccentric phase

Variable	Dominant	Non-dominant	Diff	AI%	p value	Effect size
Ankle angles (deg)						
DorsiF (+)/PlantF(−)	11.1 ± 7.6	12.0 ± 7.3	0.9	8	0.28	0.41
Ever(+)/ Inv(−)	5.4 ± 2.4	4.5 ± 2.7	0.9	17	0.39	0.33
IntR(+)/ExtR(−)	−33.5 ± 13.2	−29.1 ± 12.4	4.4	14	0.37	0.35
Ankle moments (Nm/kg)						
PlantF(+)/DorsiF(−)	1.9 ± 0.4	2.0 ± 0.4	0.1	5	0.59	0.21
Ever(+)/ Inv(−)	0.7 ± 0.2	0.7 ± 0.1	0.0	0	0.91	0.04
IntR(+)/ExtR(−)	0.1 ± 0.1	0.2 ± 0.1 *	0.1	67	0.04	0.74
Knee angles (deg)						
Flex(+)/Ext(−)	57.4 ± 6.0	60.3 ± 10.2	2.9	5	0.37	0.35
Var(+)/Valg(−)	−7.5 ± 5.0	−6.1 ± 7.1	1.4	21	0.54	0.23
IntR(+)/ ExtR(−)	21.2 ± 9.4	24.7 ± 10.5	3.5	15	0.36	0.35
Knee moments (Nm/kg)						
Ext (+)/Flex(−)	2.6 ± 0.5	2.5 ± 0.6	0.1	4	0.84	0.08
Valg(+)/Var(−)	−2.5 ± 1.0	−2.3 ± 0.8	0.2	8	0.55	0.23
IntR(+)/ExtR(−)	0.4 ± 0.1	0.3 ± 0.2	0.1	29	0.23	0.46
Hip angles (deg)						
Flex(+)/Ext(−)	45.1 ± 11.9	49.4 ± 15.9	4.3	9	0.42	0.31
Add(+)/ Ab(−)	−17.9 ± 6.7	−18.0 ± 7.6	0.1	1	0.96	0.02
IntR(+)/ExtR(−)	22.4 ± 10.1	27.2 ± 12.5	4.8	20	0.27	0.42
Hip moments (Nm/kg)						
Ext (+)/Flex(−)	4.0 ± 1.4	4.5 ± 1.6	0.5	12	0.34	0.37
Ab(+)/Add(−)	−3.6 ± 1.4	−3.3 ± 1.3	0.3	9	0.61	0.20
IntR(+)/ExtR(−)	1.3 ± 0.5	1.2 ± 0.5	0.1	8	0.91	0.04
Pelvis angles (deg)						
AntT(+)/PostT(−)	2.2 ± 5.1	3.7 ± 7.5	1.5	49	0.56	0.23
Contra Drop(+)/Contra Lift(−)	15.0 ± 5.9	14.4 ± 7.8	0.6	4	0.81	0.09
IntR(+)/ExtR(−)	−11.1 ± 13.1	−11.2 ± 12.3	0.1	1	0.98	0.01
Thorax angles (deg)						
Flex(+)/Ext(−)	30.5 ± 5.8	28.5 ± 6.4	2.0	7	0.41	0.32
LatFlex(+)/MedFlex(−)	21.0 ± 7.9	21.8 ± 5.5	0.8	4	0.75	0.12
ExtR(+)/ IntR(−)	−11.8 ± 6.6	−11.6 ± 5.6	0.2	2	0.93	0.03
Ground reaction forces (N/kg)						
Vertical	15.1 ± 2.9	16.9 ± 4.4	1.8	11	0.21	0.48
Medial/lateral	1.3 ± 0.8	1.5 ± 1.1	0.2	14	0.52	0.25
Longitudinal	9.5 ± 1.7	10.2 ± 2.7	0.7	7	0.42	0.31
Timing (s)						
Ground contact time	0.32 ± 0.04	0.35 ± 0.06	0.03	9	0.11	0.6

*Significant inter-limb difference (*p* < 0.05)

Diff: difference; AI: asymmetry index; Sig: significance

DorsiF: dorsiflexion; PlantF: plantarflexion; Ever: eversion; Inv: inversion; IntR: internal rotation; ExtR: external rotation; Flex: flexion; Ext: extension; Var: varus; Val: valgus; Add: adduction; Ab: abduction; AntT: anterior tilt; PostT: posterior tilt; Contra: contralateral; LatFlex: lateral flexion; MedFlex: medial flexion

Table 4 summarises the three variables that did display statistically significant (*p* < 0.05) asymmetries in the discrete point analysis. Two differences were associated with the pelvis, one in the drop landing and one in the hurdle hop. There was significantly greater pelvis contralateral hip lift (*p* < 0.05) when landing on the dominant leg during the drop landing. When landing on the non-dominant leg during the hurdle hop, there was significantly (*p* < 0.05) greater pelvis contralateral drop. In the cut, ankle internal rotation moments were significantly (*p* < 0.05) greater on the non-dominant side during the eccentric phase.

**Table 4:** Significant inter-limb differences (*p* < 0.05) as identified in the discrete point analysis

	Dominant Mean (±SD)	Non-dominant Mean (±SD)	Difference	*p* value	Effect size	AI%
Drop landing						
Pelvis contralateral drop(+)/lift(−) (deg)	−12.1 (4.0)	−8.9 (3.4)	3.2 (D > ND)	0.02	0.80	31
Hurdle Hop						
Pelvis contralateral drop(+)/lift(−) (deg)	−1.4 (4.7)	3.1 (4.1)	4.5 (ND > D)	0.01	0.92	264
Cut						
Ankle internal rotation moment (Nm/kg)	0.1(0.1)	0.2 (0.1)	0.1 (ND > D)	0.04	0.74	67

AI: asymmetry index; D: dominant; ND: non-dominant

**Fig. 2:**
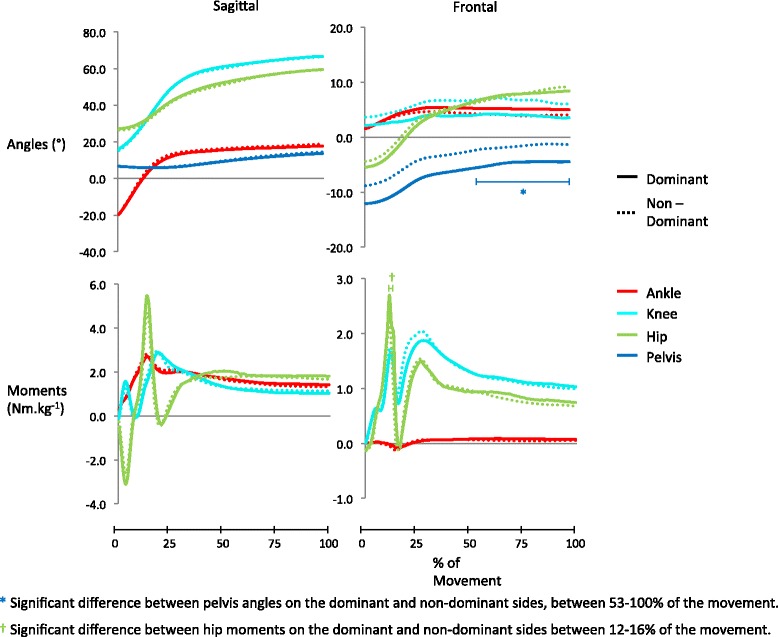
Group mean wave-forms for kinetic and kinematic variables in the drop landing. Sagittal angles: ankle dorsiflexion (+)/plantarflexion (−); knee flexion (+)/extension (−); hip flexion (+)/extension (−); pelvis anterior tilt (+)/posterior tilt(−). Frontal angles: ankle eversion (+)/inversion (−); knee varus (+)/valgus (−); hip adduction (+)/abduction (−); pelvis contralateral drop (+)/contralateral lift (−). Sagittal moments: ankle plantarflexion (+)/dorsiflexion (−); knee extension (+)/flexion (−); hip extension (+)/flexion (−). Frontal moments: ankle eversion (+)/inversion (−); knee valgus (+)/varus (−); hip abduction (+)/ adduction (−)

**Fig. 3:**
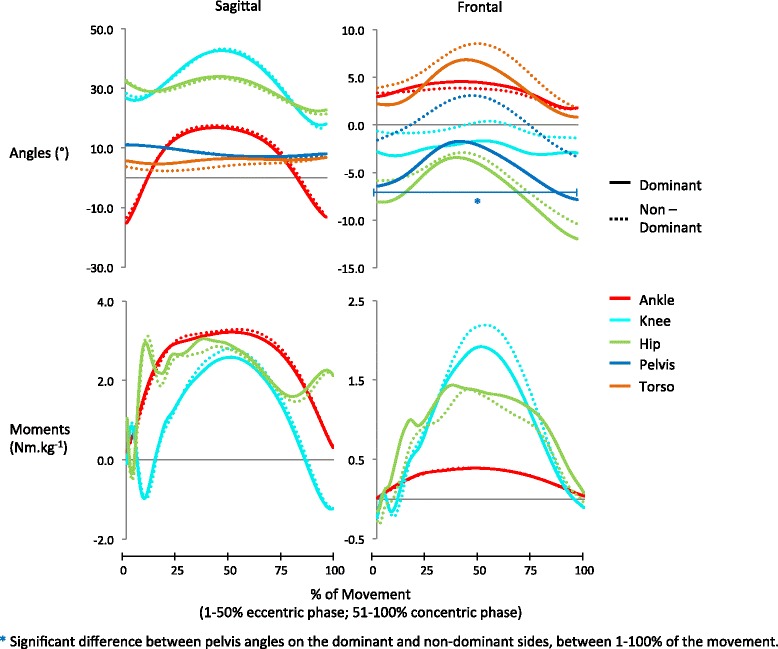
Group mean wave-forms for kinetic and kinematic variables in the hurdle hop. Sagittal angles: ankle dorsiflexion (+)/plantarflexion (−); knee flexion (+)/extension (−); hip flexion (+)/extension (−); pelvis anterior tilt (+)/posterior tilt(−); thorax flexion (+)/thorax extension (−). Frontal angles: ankle eversion (+)/inversion (−); knee varus (+)/valgus (−); hip adduction (+)/abduction (−); pelvis contralateral drop (+)/contralateral lift (−); thorax lateral flexion (+)/ medial flexion (−) Sagittal moments: ankle plantarflexion (+)/dorsiflexion (−); knee extension (+)/flexion (−); hip extension (+)/flexion (−). Frontal moments: ankle eversion (+)/inversion (−); knee valgus (+)/varus (−); hip abduction (+)/ adduction (−)

**Fig. 4:**
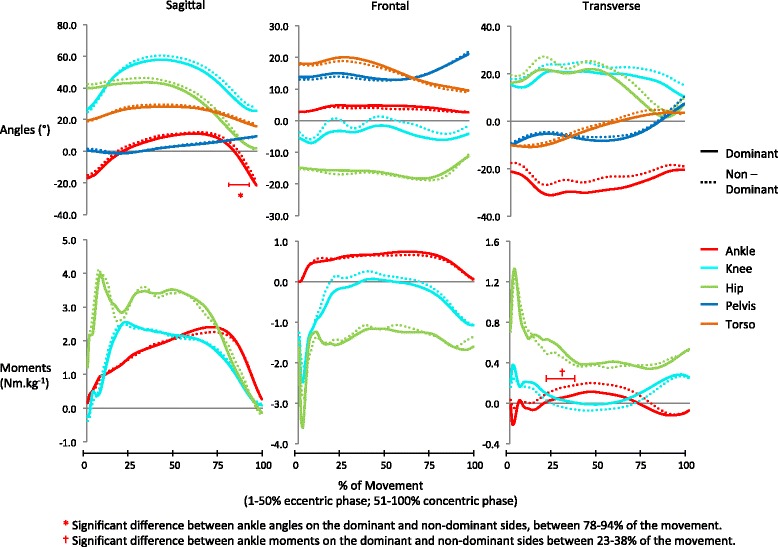
Group mean wave-forms for kinetic and kinematic variables in the cut. Sagittal angles: ankle dorsiflexion (+)/plantarflexion (−); knee flexion (+)/extension (−); hip flexion (+)/extension (−); pelvis anterior tilt (+)/posterior tilt(−); thorax flexion (+)/thorax extension (−). Frontal angles: ankle eversion (+)/inversion (−); knee varus (+)/valgus (−); hip adduction (+)/abduction (−); pelvis contralateral drop (+)/contralateral lift (−); thorax lateral flexion (+)/ medial flexion (−). Transverse angles: ankle internal rotation (+)/ external rotation(−); knee internal rotation(+)/ external rotation(−); hip internal rotation (+)/ hip external rotation (−); pelvis internal rotation(+)/ external rotation(−); thorax external rotation (+)/internal rotation (−). Sagittal moments: ankle plantarflexion (+)/dorsiflexion (−); knee extension (+)/flexion (−); hip extension (+)/flexion (−). Frontal moments: ankle eversion (+)/inversion (−); knee valgus (+)/varus (−); hip abduction (+)/ adduction (−). Transverse moments: ankle internal rotation (+)/external rotation (−); knee internal rotation (+)/external rotation(−); hip internal rotation(+)/external rotation (−)

**Fig. 5:**
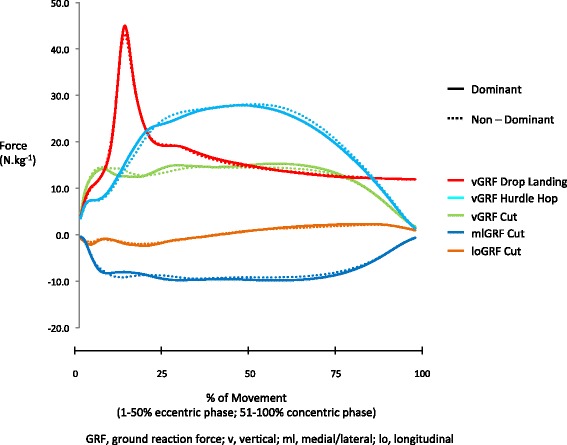
Group mean wave forms for ground reaction forces in the drop landing, hurdle hop and cut

For the ACP, Figs. 2, 3, 4 and 5 display group mean wave-forms for all variables in the drop landing, hurdle hop and cut, respectively. Areas of the wave-form that displayed significant differences between dominant and non-dominant leg are highlighted. The majority of variables under examination displayed no significant asymmetries in the drop landing (13/15), hurdle hop (16/17) or cut (26/28). Those variables that did display significant differences (*p* < 0.05) are summarised in Table 5. For the drop landing on the dominant leg there was significantly greater hip abductor moments early in the eccentric phase (*p* = 0.02, effect size = 0.62) and more pelvis contralateral lift from 52 % of the movement onwards (*p* = 0.04, effect size = 0.66). There was significantly greater contralateral pelvic drop on the non-dominant side throughout the hop test (*p* = 0.01 - 0.02, effect size = 0.88). In the cut, ankle internal rotation moments were significantly greater in the non-dominant ankle (*p* = 0.02 – 0.04, effect size = 0.52) from 23-38 % of the movement. The ankle joint was also significantly more dorsi-flexed on the non-dominant side during the latter stages (78–94 %) of the cut push-off (*p* = 0.011, effect size = 0.57).

**Table 5:** Significant inter-limb differences (*p* < 0.05) as identified in the analysis of characterising phases

Variable	Difference	Percentage of movement (%)	*p* value	Effect size
Drop landing				
Hip abductor moment (Nm/kg)	D > ND	12-16	0.02	0.62
Pelvis contralateral lift (deg)	D > ND	53-100	0.04	0.66
Hurdle Hop				
Pelvis contralateral drop (deg)	ND > D	1 - 100	0.02	0.88
Cut				
Ankle internal rotation moment (Nm/kg)	ND > D	23-38	0.04	0.52
Ankle dorsiflexion (deg)	ND > D	78 - 94	0.01	0.57

D: dominant; ND: non-dominant

The test-retest reliability findings for variables in the drop landing, hurdle hop and cut are detailed in Additional file 6: Table S3. There were no significant differences in reliability scores between limbs so the values provided in Additional file 6: Table S3 are the mean ICC values of the dominant and non-dominant sides. All variables displayed good to excellent reliability (ICC > 0.60) in the drop landing (mean ICC [95 % confidence intervals (CI)]: 0.89 [0.90, 0.88]), hurdle hop (0.88 [0.89, 0.87]), and cut (0.85 [0.86, 0.84]).

## Discussion

Our findings highlighted a clear tendency toward biomechanical inter-limb symmetry during multi directional neuromuscular challenge tests in a cohort of elite, injury free, rugby union players. Asymmetries that were identified were limited to frontal plane pelvis angles and moments in the drop landing and hurdle hop, alongside ankle sagittal plane angle and internal rotation moment in the cut. The analysis of characterising phases (ACP) identified two additional asymmetries not identified in the discrete point analysis. Previous investigations of symmetry in elite athletes have utilised tests such as isokinetic dynamometry [40] but these are uni-planar assessments of a single joint, which do not have immediate relevance to athletic movement. Conversely, studies that have examined more dynamic tasks like running have done so only in linear running at a submaximal pace or with sub-elite athletes [6].

Hip eccentric abductor moment in the drop landing and ankle dorsiflexion angle in the cut (Tables 4 and 5) were found to be asymmetrical in the ACP, but not in the discrete point analysis. It would appear that these asymmetries were missed in the discrete analysis because the phase of the movement where the difference lay did not coincide with their peak magnitude (Figs. 2 and 4). Similar to work by Richter and colleagues [37] and Shorter and colleagues [41], our findings highlight the benefit of using continuous movement plane analysis techniques when examining biomechanical data as they do not require *a priori* knowledge of which event/phase to analyse.

While the majority of variables exhibited no significant asymmetry, several exhibited a large asymmetry index (AI) in the discrete point analysis; AI ranges for symmetrical variables in the drop, hop and cut were 0–143 %, 0–264 % and 0–49 %, respectively (Tables 1–3). These differences are likely due to the AI calculation being overly sensitive to variables with small magnitudes and tending to inflate their score as a result [24]. In the drop landing, for example, knee varus angle and knee flexion angle differed by similar amounts between dominant and non-dominant legs (3° and 2°, respectively), but the AIs for these variables were notably different (143 % and 3 %, respectively). This is due to the magnitudes of knee varus being approximately ten times smaller than the magnitudes of knee flexion (Table 1). It appears that frontal plane variables in the drop and hop are particularly affected by the inflation of AI scores due to small variable magnitudes (Tables 1 and 2). If frontal plane variables are excluded, ranges of AI fall to 0–31 % in the drop landing and 0–7 % in the hurdle hop which are closer to the 0–49 % in the cut and the 3–50 % found in studies of straight line running [6]. These findings, which are similar to those of Herzog and colleagues [24] in gait analysis, suggest that the use of AIs to provide normative symmetry values for biomechanical variables of small magnitude (e.g. knee varus/valgus) is questionable. As an alternative it may be more appropriate to simply examine magnitude differences between limbs for each variable of interest. To this end the results presented in Tables 1–3 for discrete points, and in Figs. 2–4 for the complete movement phase, provide useful normative values for rehabilitation specialists who are undertaking injury screening testing or monitoring rehabilitation progress in similar population groups.

In total, five variables were found to display significant inter-limb asymmetries. Pelvis contralateral lift and hip eccentric abductor moment in the drop landing were greater on the dominant side, while pelvis contralateral drop in the hurdle hop, ankle eccentric internal rotation moment and ankle dorsiflexion angle in the cut were all greater on the non-dominant side (Tables 4 and 5). It would appear that in the drop landing, participants were able to generate larger eccentric hip abductor moments on the dominant leg early in the landing (Table 5) which allowed them to achieve a greater contralateral pelvis lift later in the movement (Table 5). This may be as a result of a different landing strategy on the dominant side as a result of preferential use in training [26, 42]. Vittasalo and colleagues [26] found that training history influences the timing and magnitude of lower extremity muscle activation on landing in a jump. They found that trained athletes activated their lower extremity muscles earlier and to a greater extent than physically active controls [26].

Preferential use of the dominant limb during training may also explain, at least in part, the asymmetries observed in the hurdle hop, a movement which places an emphasis on frontal plane movement control. Participants exhibited a significant contralateral pelvis drop on the non-dominant limb but in contrast maintained a contralateral lift throughout the movement on the dominant limb (Fig. 3). This particular asymmetry had the largest effect size of all significant findings (discrete analysis = 0.93; ACP = 0.88), and was present throughout the entire movement phase (Table 5 and Fig. 3). A contralateral pelvis drop on the non-dominant leg may be as a result of poorer neuromuscular control produced by the hip abductors (e.g. gluteus medius) [43–46] and may indicate a reduced ability to protect the knee from the excessive frontal plane moments associated with injury [13].

In the cut, the non-dominant side exhibited significantly greater ankle eccentric internal rotation moments early in the movement (Tables 5) and a more dorsiflexed/less plantar flexed ankle during the later phase of the movement (Table 5 and Fig. 4). Further examination of the data identified a highly significant correlation (*r* = 0.86, *p* < 0.01) between these variables indicating that the greater ankle internal rotation moments are related to the greater ankle dorsiflexion/less plantarflexion. The actual relevance of these asymmetries in elite athletes from an injury development standpoint, as with all of the asymmetries discussed here, requires further investigation with prospective studies. In addition, it is important to emphasise that while our findings illustrate that in an uninjured group of elite players some dominant versus non-dominant asymmetries may exist, the vast majority of variables exhibited no significant asymmetries. This provides a very valuable set of normative data with which to examine whether asymmetries in individuals are indicative of a predisposition to injury.

While the current study provides useful normative data for the movements examined, it is accepted as a limitation that the sample size was of twenty single-sport multidirectional athletes. A replication of this study with a larger number of participants, and with players from different sports, would enhance the knowledge base beyond this study. A potential limitation of the current study is that the neuromuscular challenge tests examined were all pre-planned, with no indecision element. It may be argued that movement in response to a sudden stimulus may elicit different and more sport specific movement patterns and thus may potentially provide a greater test of symmetry [47, 48]. Based on findings from a meta-analysis undertaken by Brown and colleagues [49], substantial increases in frontal plane knee abductor moments (approximately 63 %) and knee internal rotator moments (up to 127 %) may be expected when undertaking un-planned in comparison to pre-planned cuts. Knee angles in all three movement planes would also be expected to increase [49]. Increases such as this could facilitate the identification of asymmetries that may be masked in less challenging pre-planned cuts.

## Conclusions

Elite, injury free, rugby union players tend to exhibit bi-lateral symmetry across a broad range of biomechanical variables in a single leg drop landing, a single leg hurdle hop and a cutting manoeuvre. This study provides useful normative values for inter-limb symmetry in these movement tests. In addition it is recommended to utilise data analysis techniques that allow an examination of continuous data as opposed to discrete points; a discrete point analysis was unable to detect two of the five asymmetries identified. Our findings highlighted that the use of an asymmetry index as a standard measure of symmetry in biomechanical variables is questionable due to its sensitivity to variable magnitude. Asymmetries identified in this study were limited to frontal plane pelvis angles and moments in the drop landing and hurdle hop, alongside ankle sagittal plane angles and internal rotation moment in the cut. Prospective studies are required to establish the relevance of these biomechanical asymmetries in the development of injuries.

## Additional files

Additional file 4: Table S1. Hurdle hop discrete point findings—inter-limb differences in peak variable magnitudes during the concentric phase. Inter-limb differences in peak variable magnitudes during the concentric phase of the hurdle hop movement. Additional file 5: Table S2. Running cut discrete point findings—inter-limb differences in peak variable magnitudes during the concentric phase. Inter-limb differences in peak variable magnitudes during the concentric phase of the running cut movement. Additional file 6: Table S3. Intraclass correlation coefficient (test-retest reliability) of measures in the drop landing, hurdle hop and cut. Test-retest reliability scores of measures in the drop landing, hurdle hop and cut.

## Abbreviations

ACP: Analysis of characterising phases
3D: Three dimensional
AI: Asymmetry index
