# Supplementary material for: Biomechanical symmetry in elite rugby union players during dynamic tasks: an investigation using discrete and continuous data analysis techniques
Source: BMC Sports Sci Med Rehabil. 2015 Jun 19;7:13. doi: 10.1186/s13102-015-0006-9 (PMC4940714; doi:10.1186/s13102-015-0006-9)
Supplement: Additional file 4: Table S1. — Hurdle hop discrete point findings—inter-limb differences in peak variable magnitudes during the concentric phase. Inter-limb differences in peak variable magnitudes during the concentric phase of the hurdle hop movement. [file 13102_2015_6_MOESM4_ESM.docx]

Table S1. Hurdle hop discrete point findings – inter-limb differences in peak variable magnitudes during the concentric phase

| Variable | Dominant | Non-dominant | Diff | AI% | p value | Effect size |
| --- | --- | --- | --- | --- | --- | --- |
| **Ankle angles (deg)** |  |  |  |  |  |  |
| DorsiF (+)/PlantF (-) | 16.8 ± 4.3 | 17.6 ± 4.5 | 0.8 | 5 | 0.62 | 0.19 |
| Ever(+)/ Inv(-) | 4.2 ± 2.4 | 3.7 ± 2.2 | 0.5 | 12 | 0.60 | -0.20 |
| **Ankle moments (Nm/kg)** |  |  |  |  |  |  |
| PlantF(+)/DorsiF (-) | 3.3 ± 0.6 | 3.3 ± 0.5 | 0.0 | 1 | 0.87 | 0.06 |
| Ever(+)/ Inv(-) | 0.4 ± 0.2 | 0.4 ± 0.2 | 0.0 | 1 | 0.97 | -0.01 |
| **Knee angles (deg)** |  |  |  |  |  |  |
| Flex(+)/Ext (-) | 42.8 ± 9.9 | 43.8 ± 8.9 | 1.0 | 2 | 0.79 | 0.11 |
| Var(+)/Valg(-) | -3.3 ± 5.5 | -0.9 ± 4.4 | 2.4 | 117 | 0.21 | 0.48 |
| **Knee moments (Nm/kg)** |  |  |  |  |  |  |
| Ext (+)/Flex(-) | 2.7 ± 0.7 | 2.9 ± 0.5 | 0.2 | 7 | 0.39 | 0.33 |
| Valg(+)/Var(-) | 2.0 ± 0.6 | 2.3 ± 0.5 | 0.3 | 14 | 0.15 | 0.55 |
| **Hip angles (deg)** |  |  |  |  |  |  |
| Flex(+)/Ext (-) | 31.6 ± 9.0 | 30.6 ± 8.2 | 1.0 | 3 | 0.75 | -0.12 |
| Add(+)/ Ab(-) | -11.8 ± 6.2 | -9.7 ± 5.5 | 2.1 | 19 | 0.37 | 0.35 |
| **Hip moments (Nm/kg)** |  |  |  |  |  |  |
| Ext (+)/Flex(-) | 2.7 ± 0.8 | 2.5 ± 0.5 | 0.2 | 8 | 0.66 | -0.17 |
| Ab(+)/Add(-) | 1.4 ± 0.3 | 1.3 ± 0.4 | 0.1 | 8 | 0.38 | 0.34 |
| **Pelvis angles (deg)** |  |  |  |  |  |  |
| AntT(+)/PostT(-) | 9.5 ± 4.1 | 8.6 ± 3.1 | 0.9 | 10 | 0.52 | -0.25 |
| Contra Drop(+)/  Contra Lift(-) | -1.7 ± 4.9 | 3.1 ± 4.4 * | 4.8 | 693 | 0.01 | 0.94 |
| **Thorax angles (deg)** |  |  |  |  |  |  |
| Flex(+)/Ext(-) | -7.9 ± 8.2 | -6.7 ± 7.7 | 1.2 | 17 | 0.68 | 0.16 |
| LatFlex(+)/  MedFlex(-) | 7.6 ± 5.9 | 8.8 ± 4.5 | 1.2 | 14 | 0.57 | 0.22 |

- Significant inter-limb difference (p<0.05)

Diff: difference; AI: asymmetry index; Sig: significance.

DorsiF: dorsiflexion; PlantF: plantarflexion; Ever: eversion; Inv: inversion; Flex: flexion; Ext: extension; Var: varus; Val: valgus; Add: adduction; Ab: abduction; AntT: anterior tilt; PostT: posterior tilt; Contra: contralateral; LatFlex: lateral flexion; MedFlex: medial flexion.
