# Supplementary material for: Biomechanical symmetry in elite rugby union players during dynamic tasks: an investigation using discrete and continuous data analysis techniques
Source: BMC Sports Sci Med Rehabil. 2015 Jun 19;7:13. doi: 10.1186/s13102-015-0006-9 (PMC4940714; doi:10.1186/s13102-015-0006-9)
Supplement: Additional file 5: Table S2. — Running cut discrete point findings—inter-limb differences in peak variable magnitudes during the concentric phase. Inter-limb differences in peak variable magnitudes during the concentric phase of the running cut movement. [file 13102_2015_6_MOESM5_ESM.docx]

Table S2 Running cut discrete point findings – inter-limb differences in peak variable magnitudes during the concentric phase

| Variable | Dominant | Non-dominant | Diff | AI% | p value | Effect size |
| --- | --- | --- | --- | --- | --- | --- |
| **Ankle angles (deg)** |  |  |  |  |  |  |
| DorsiF (+)/PlantF (-) | -21.6 ± 5.8 | -18.6 ± 7.6 | 3.0 | 15 | 0.26 | 0.44 |
| Ever(+)/ Inv(-) | 5.2 ± 2.8 | 4.4 ± 2.7 | 0.8 | 16 | 0.45 | -0.29 |
| IntR(+)/ExtR(-) | -31.9 ± 14.0 | -28.4 ± 11.5 | 3.5 | 12 | 0.48 | 0.27 |
| **Ankle moments (Nm/kg)** |  |  |  |  |  |  |
| PlantF(+)/DorsiF (-) | 2.5 ± 0.3 | 2.5 ± 0.3 | 0.0 | 0 | 1.00 | 0.00 |
| Ever(+)/ Inv(-) | 0.8 ± 0.2 | 0.7 ± 0.1 | 0.1 | 13 | 0.40 | -0.32 |
| IntR(+)/ExtR(-) | -0.2 ± 0.1 | -0.2 ± 0.1 | 0.0 | 0 | 0.76 | -0.12 |
| **Knee angles (deg)** |  |  |  |  |  |  |
| Flex(+)/Ext (-) | 58.0 ± 6.5 | 61.3 ± 9.9 | 3.3 | 5 | 0.31 | 0.39 |
| Var(+)/Valg(-) | -5.7 ± 5.9 | -4.6 ± 7.0 | 1.1 | 21 | 0.66 | 0.17 |
| IntR(+)/ ExtR(-) | 20.5 ± 10.4 | 23.2 ± 10.4 | 2.7 | 13 | 0.49 | 0.27 |
| **Knee moments (Nm/kg)** |  |  |  |  |  |  |
| Ext (+)/Flex(-) | 2.2 ± 0.6 | 2.1 ± 0.7 | 0.1 | 5 | 0.78 | -0.11 |
| Valg(+)/Var(-) | -0.8 ± 0.4 | -0.9 ± 0.3 | 0.1 | 12 | 0.43 | 0.30 |
| ExtR(+)/IntR(-) | 0.2 ± 0.1 | 0.2 ± 0.1 | 0.0 | 0 | 0.77 | -0.11 |
| **Hip angles (deg)** |  |  |  |  |  |  |
| Flex(+)/Ext (-) | 42.6 ± 13.9 | 46.6 ± 17.1 | 4.0 | 9 | 0.50 | 0.26 |
| Add(+)/ Ab(-) | -19.9 ± 5.9 | -21.2 ± 7.6 | 1.3 | 6 | 0.63 | -0.18 |
| IntR(+)/ExtR(-) | 19.5 ± 9.7 | 24.3 ± 11.8 | 4.8 | 22 | 0.25 | 0.44 |
| **Hip moments (Nm/kg)** |  |  |  |  |  |  |
| Ext (+)/Flex(-) | 3.9 ± 0.9 | 3.7 ± 0.7 | 0.3 | 8 | 0.44 | 0.30 |
| Ab(+)/Add(-) | -1.1 ± 0.3 | -1.1 ± 0.6 | 0.0 | 0 | 0.93 | -0.03 |
| IntR(+)/ExtR(-) | 0.4 ± 0.1 | 0.4 ± 0.2 | 0.0 | 0 | 0.28 | 0.41 |
| **Pelvis angles (deg)** |  |  |  |  |  |  |
| AntT(+)/PostT(-) | 9.6 ± 5.0 | 9.2 ± 7.6 | 0.4 | 4 | 0.89 | -0.06 |
| Contra Drop(+)/  Contra Lift(-) | 19.9 ± 3.9 | 20.0 ± 5.2 | 0.1 | 0 | 0.98 | 0.01 |
| IntR(+)/ExtR(-) | 7.5 ± 11.1 | 10.5 ± 12.6 | 3.0 | 33 | 0.51 | 0.25 |
| **Thorax angles (deg)** |  |  |  |  |  |  |
| Flex(+)/Ext(-) | 28.9 ± 6.3 | 30.6 ± 5.6 | 1.7 | 6 | 0.44 | -0.30 |
| LatFlex(+)/  MedFlex(-) | 19.2 ± 8.0 | 18.6 ± 5.2 | 0.6 | 3 | 0.83 | -0.08 |
| ExtR(+)/ IntR(-) | -5.6 ± 4.3 | -6.6 ± 5.2 | 1.0 | 15 | 0.61 | -0.20 |

Diff: difference; AI: asymmetry index; Sig: significance.

DorsiF: dorsiflexion; PlantF: plantarflexion; Ever: eversion; Inv: inversion; IntR: internal rotation; ExtR: external rotation; Flex: flexion; Ext: extension; Var: varus; Val: valgus; Add: adduction; Ab: abduction; AntT: anterior tilt; PostT: posterior tilt; Contra: contralateral; LatFlex: lateral flexion; MedFlex: medial flexion.
