# Supplementary material for: Biomechanical symmetry in elite rugby union players during dynamic tasks: an investigation using discrete and continuous data analysis techniques
Source: BMC Sports Sci Med Rehabil. 2015 Jun 19;7:13. doi: 10.1186/s13102-015-0006-9 (PMC4940714; doi:10.1186/s13102-015-0006-9)
Supplement: Additional file 6: Table S3. — Intraclass correlation coefficient (test-retest reliability) of measures in the drop landing, hurdle hop and cut. Test-retest reliability scores of measures in the drop landing, hurdle hop and cut. [file 13102_2015_6_MOESM6_ESM.docx]

Table S3 Intraclass correlation coefficient (test-retest reliability) of measures in the drop landing, hurdle hop and cut

| Variable | Drop landing | Hurdle Hop | Cut |
| --- | --- | --- | --- |
|  |  |  |  |
| **Ankle angles (deg)** |  |  |  |
| DorsiF /PlantF | 0.93 | 0.89 | 0.77 |
| Ever/ Inv | 0.92 | 0.88 | 0.89 |
| IntR/ExtR | 0.93 | 0.93 | 0.88 |
| **Ankle moments (Nm/kg)** |  |  |  |
| PlantF/DorsiF | 0.94 | 0.90 | 0.76 |
| Ever/ Inv | 0.93 | 0.67 | 0.61 |
| IntR/ExtR | 0.92 | 0.85 | 0.73 |
| **Knee angles (deg)** |  |  |  |
| Flex/Ext | 0.95 | 0.91 | 0.60 |
| Var/Valg | 0.95 | 0.93 | 0.90 |
| IntR/ ExtR | 0.91 | 0.89 | 0.91 |
| **Knee moments (Nm/kg)** |  |  |  |
| Ext/Flex | 0.82 | 0.89 | 0.90 |
| Valg/Var | 0.87 | 0.83 | 0.86 |
| IntR/ExtR | 0.80 | 0.89 | 0.96 |
| **Hip angles (deg)** |  |  |  |
| Flex/Ext | 0.90 | 0.88 | 0.92 |
| Add/ Ab | 0.92 | 0.89 | 0.91 |
| IntR/ExtR | 0.88 | 0.97 | 0.95 |
| **Hip moments (Nm/kg)** |  |  |  |
| Ext/Flex | 0.86 | 0.93 | 0.88 |
| Ab/Add | 0.94 | 0.86 | 0.69 |
| IntR/ExtR | 0.85 | 0.75 | 0.92 |
| **Pelvis angles (deg)** |  |  |  |
| AntT/PostT | 0.91 | 0.88 | 0.91 |
| Contra Drop/  Contra Lift | 0.62 | 0.79 | 0.88 |
| IntR/ExtR | 0.91 | 0.96 | 0.79 |
| **Thorax angles (deg)** |  |  |  |
| Flex(+)/Ext(-) | . | 0.93 | 0.96 |
| LatFlex(+)/  MedFlex(-) | . | 0.84 | 0.92 |
| ExtR(+)/ IntR(-) | . | 0.91 | 0.79 |
